# Anxiety, depression, and their association with unintentional injury risk among older adult populations in Guangxi, China: a cross-sectional study

**DOI:** 10.3389/fpubh.2024.1466083

**Published:** 2025-01-14

**Authors:** Li Niu, Jie Zhang, Chun-liu Lu, Yong Zhang, Xin-xin Mo, Rong Xu, Hong-ye Luo

**Affiliations:** ^1^Department of Preventive Medicine, School of Basic Medical Sciences, Jiujiang University, Jiujiang, Jiangxi, China; ^2^Center for Health Statistics and Information, Health Commission of Guangxi, Nanning, Guangxi, China; ^3^Health Management Center, Jiangbin Hospital, Nanning, Guangxi, China; ^4^Department of Gastroenterology, The Eighth People's Hospital of Nanning, Nanning, Guangxi, China; ^5^Research Centre for Regenerative Medicine, Guangxi Medical University, Nanning, Guangxi, China; ^6^Organization Department of the Party Committee, Guangxi Medical University, Nanning, Guangxi, China; ^7^Department of Health Care Management, School of Information and Management, Guangxi Medical University, Nanning, Guangxi, China

**Keywords:** unintentional injuries, older adult population, mental health, anxiety and depression, rural health disparities

## Abstract

**Background:**

The aging population presents a significant public health challenge, particularly concerning mental health and injury prevention. Anxiety and depression are common among the older adult, affecting their quality of life and increasing the risk of unintentional injuries (UI). This study aims to explore the association between anxiety and depression and UI risk among the older adult in Guangxi, China, using data from the 2023 National Health Service Survey.

**Methods:**

A cross-sectional design was employed, analyzing data from 2,894 participants aged 60 and above in Guangxi. The primary variables were anxiety and depression, assessed via validated scales, with UI as the dependent variable. Logistic regression was used to estimate crude and adjusted odds ratios (OR) with 95% confidence intervals (CI), adjusting for potential confounders such as age, gender, socioeconomic status, and lifestyle factors.

**Results:**

Significant findings indicate that individuals with anxiety and depression had nearly double the risk of UI compared to those without these conditions (adj. OR = 1.92, 95% CI: 1.42–2.6, *p* < 0.001). Alcohol consumption was also associated with higher UI risk (adj. OR = 1.46, 95% CI: 1.05–2.03, *p* = 0.023). Females had a significantly higher adjusted odds of UI compared to males (adj. OR = 1.38, 95% CI: 1.03–1.84, *p* = 0.029), and rural residents were more likely to experience UI than urban dwellers (adj. OR = 1.38, 95% CI: 1.05–1.82, *p* = 0.02). Exercise frequency was inversely related to UI risk, with those exercising 1–2 times per week having reduced odds (adj. OR = 0.46, 95% CI: 0.22–0.98, *p* = 0.044). Other factors such as age, marital status, hypertension, diabetes, and main caregiver showed no significant associations with UI.

**Conclusion:**

Addressing mental health issues and promoting moderate exercise may help reduce UI risk in the older adult. Policies should focus on enhancing mental health services and injury prevention programs, particularly in rural settings, to improve the overall health and safety of the aging population in Guangxi.

## 1 Introduction

The aging population globally presents a complex challenge to public health systems, particularly in terms of mental health and injury prevention. Anxiety and depression are among the most common mental health disorders affecting the older adult, significantly influencing their quality of life and physical health (1). Research has consistently shown that both anxiety and depression are linked to increased risks of physical health issues, including Unintentional Injuries (UI) (2). UI refer to injuries that occur without intent to harm, such as falls, burns, or accidents, and are distinct from traumatic injuries, which may result from intentional acts or violence (3). UI among the older adult remain a leading cause of morbidity and mortality, often leading to substantial long-term impacts on health and significant healthcare costs (4). Study showed that the prevalence of UI in rural older adult population was 44.4%, and participants aged 75–84 years had the highest rates (48.8%) (5). The relationship between mental health disorders like anxiety and depression and the risk of injuries is particularly pronounced in this demographic due to factors such as reduced mobility, poorer balance, and cognitive impairments that can accompany aging (6).

While the association between mental health disorders and increased risk of unintentional injuries in the older adult is documented globally, specific data from the Guangxi region of China remains sparse. The region’s unique demographic composition, characterized by a high proportion of aging rural residents and distinct socio-economic conditions (7). Rural settings often lack adequate mental health services, which can exacerbate the consequences of untreated anxiety and depression, potentially leading to a higher incidence of injuries (8, 9). Current literature predominantly centers on urban populations or those in more developed regions, leaving a significant knowledge gap regarding rural and economically diverse areas like Guangxi. Research in Guangxi has not yet comprehensively addressed how these mental health conditions affect the older adult, particularly in terms of their physical safety (10). Studies have highlighted the growing prevalence of mental health issues in older adult Chinese populations but have not delved into the specific consequences of these issues on physical health outcomes, such as injuries, in less urbanized areas (11, 12).

This research aims to bridge this gap by utilizing the data from the 2023 National Health Service Survey to explore the association between anxiety and depression and the risk of unintentional injuries among the older adult in Guangxi. By doing so, it seeks to provide targeted insights that can guide local health policies and interventions tailored to the unique needs of Guangxi’s aging rural population (13).

## 2 Materials and methods

### 2.1 Study design

The current study utilizes a cross-sectional design to investigate the impact of anxiety and depression on the risk of unintentional injuries among older adult populations in Guangxi, China. Data for this study are sourced from the 2023 National Health Service Survey, which includes comprehensive health-related information from a representative sample of the Chinese population, focusing particularly on rural and urban disparities in health outcomes, healthcare access, and utilization. The survey employs a multistage, stratified, cluster sampling design to ensure representativeness of the national population, including the older adult cohort in Guangxi. The sample includes individuals aged 60 years and above, residing in both urban and rural areas of Guangxi.

The primary independent variables are indicators of anxiety and depression, assessed through validated scales included in the survey. The primary dependent variable is the occurrence of unintentional injuries reported during the survey period. Additional variables such as age, gender, socioeconomic status, urban vs. rural residence, access to healthcare, and lifestyle factors (e.g., exercise frequency, and alcohol use) are controlled for in the analysis to adjust for potential confounders.

### 2.2 Participants

The inclusion criteria for participants in this study are based on the demographic data collected through the 2023 National Health Service Survey. Participants included in the analysis meet the following criteria: Age: Individuals aged 60 years and above at the time of the survey. Residency: Residents of the Guangxi region, encompassing both urban and rural areas.

Exclusion Criteria are as follows: Individuals with missing or incomplete data on key variables of interest, such as mental health status (anxiety and depression) or reports of unintentional injuries, are excluded from the analysis. Individuals reported or diagnosed with severe cognitive impairments. Individuals who are not long-term residents of Guangxi or who were temporarily residing in the area during the survey period are excluded.

### 2.3 Data collection

The data for this study were collected as part of the 2023 National Health Service Survey, a comprehensive health survey conducted under the auspices of the National Health Commission of China. The survey is designed to gather a wide range of health-related information across various demographics and regions within China, including Guangxi.

Survey Instruments includes: The survey utilized structured questionnaires designed to collect detailed information on health status, lifestyle factors, access to healthcare services, and social determinants of health. Specific sections of the questionnaire were dedicated to assessing mental health, using standardized screening tools for anxiety and depression. The tool is EQ-5D-5L, which are widely used and validated instruments for detecting levels of anxiety and depression. The EQ-5D-5L was utilized to assess self-reported levels of anxiety and depression. It captures the presence and severity of anxiety and depressive symptoms but does not provide a clinical diagnosis. Therefore, our findings reflect symptomatology rather than clinically diagnosed mental health disorders. Information on unintentional injuries was collected through specific questions relating to any injuries the participants had sustained over the past 12 months.

Trained interviewers conducted face-to-face interviews with participants, ensuring that each individual understood the questions and provided informed responses. Responses were directly entered into electronic devices with data collection software. All interviewers underwent rigorous training, focusing on the ethical considerations of data collection, the importance of confidentiality, and the specific techniques required to accurately collect and record responses. Supervisors regularly monitored the data collection process to ensure adherence to protocols and to address any issues arising during the interviews.

### 2.4 Statistical analysis

In this study, the prevalence of anxiety and depression among the older adult population in Guangxi was calculated using percentages, while demographic and other relevant characteristics such as age, gender, socio-economic status, and rural vs. urban residence were summarized using means, standard deviations, and percentages. Inferential statistics included Chi-Square tests to examine the relationship between the presence of mental health conditions and unintentional injuries. Logistic regression was employed to assess the odds of such injuries associated with anxiety and depression, with models progressively adjusting for potential confounders. Multivariate analyses tested interaction effects to see if the impact of mental health on injury risk varied by factors such as rural vs. urban residence, and model fit was assessed using the Hosmer-Lemeshow test. Sensitivity analyses, including multiple imputation for missing data and robustness checks with different model specifications, were also conducted to ensure the reliability of the findings.

### 2.5 Ethical considerations

The survey was approved by the National Bureau of Statistics of China, and the study protocol received approval from the Medical Ethics Expert Committee of the National Health Commission of China, ensuring compliance with national and international ethical standards. Prior to participation, all participants provided informed consent, fully briefed on the study’s purpose, procedures, risks, and benefits, with assurance of their right to withdraw at any time without penalty, all communicated in a clear and understandable manner. Strict confidentiality and privacy measures were implemented, with all identifiable information anonymized and data access restricted to authorized personnel. Risk assessment determined that participation risks were minimal, but provisions were made to address any potential adverse effects, including referrals to appropriate medical or psychological resources. The authors declared no conflicts of interest, affirming that funding sources did not influence the study’s design, execution, or reporting. The team upheld cultural sensitivity to accommodate the diverse backgrounds of the Guangxi participants and committed to the standards of data integrity and responsible reporting. Findings were reported transparently, irrespective of support or contradiction to initial hypotheses. The manuscript adhered to ICMJE ethical publishing guidelines, including a thorough peer review process to validate its scientific integrity and value.

## 3 Results

The study analyzed data from a total of 2,894 older adult participants aged 60 years and above residing in Guangxi, China. Among these, 298 individuals (10.3%) reported experiencing unintentional injuries (UIs) within the past 12 months. Table 1 showed the associations between various demographic, socioeconomic, and health-related factors and the incidence of unintentional injuries (UI) in an older adult population. Significant findings include a higher likelihood of UI among those experiencing anxiety and depression [Chisq. (1 df) = 22.28, *p* < 0.001], while other factors such as age, gender, ethnicity, education level, marital status, alcohol consumption, exercise frequency, area of residence, hypertension, diabetes, monthly income, and main caregiver showed no significant associations with UI, as indicated by their respective *p*-values exceeding 0.05.

**Table 1:** Analysis of factors associated with Unintentional Injuries in older adult population.

	No UI	UI	Test stat.	*p*-value
Total	2,596	298		
Age group			Chisq. (4 df) = 3.45	0.485
60–64	621 (23.9)	63 (21.1)		
65–69	685 (26.4)	72 (24.2)		
70–74	598 (23)	70 (23.5)		
75–79	293 (11.3)	39 (13.1)		
80+	399 (15.4)	54 (18.1)		
Gender			Chisq. (1 df) = 2.18	0.14
Male	1,267 (48.8)	132 (44.3)		
Female	1,329 (51.2)	166 (55.7)		
Ethnicity			Chisq. (1 df) = 0.05	0.823
Han	1971 (75.9)	228 (76.5)		
Ethnic minority	625 (24.1)	70 (23.5)		
Education level			Chisq. (1 df) = 0.19	0.666
High School and below	2,413 (93)	279 (93.6)		
College and above	183 (7)	19 (6.4)		
Marital status			Chisq. (3 df) = 3.85	0.278
Unmarried	26 (1)	4 (1.3)		
Married	1979 (76.2)	214 (71.8)		
Widowed	555 (21.4)	73 (24.5)		
Divorced	36 (1.4)	7 (2.3)		
Anxiety and depression			Chisq. (1 df) = 22.28	< 0.001
Yes	340 (13.1)	69 (23.2)		
No	2,256 (86.9)	229 (76.8)		
Alcohol consumption			Chisq. (1 df) = 0.88	0.347
Yes	557 (21.5)	71 (23.8)		
No	2039 (78.5)	227 (76.2)		
Exercise frequency in 1 month			Chisq. (4 df) = 7.09	0.131
>6 times	918 (35.4)	100 (33.6)		
3–5 times	181 (7)	16 (5.4)		
1–2 times	141 (5.4)	8 (2.7)		
<1 time	38 (1.5)	5 (1.7)		
None	1,318 (50.8)	169 (56.7)		
Area			Chisq. (1 df) = 3.03	0.082
Rural	1,546 (59.6)	193 (64.8)		
Urban	1,050 (40.4)	105 (35.2)		
Hypertension			Chisq. (1 df) = 0.52	0.47
Yes	852 (32.8)	104 (34.9)		
No	1744 (67.2)	194 (65.1)		
Diabetes			Chisq. (1 df) = 2.57	0.109
Yes	262 (10.1)	39 (13.1)		
No	2,334 (89.9)	259 (86.9)		
Monthly income			Chisq. (3 df) = 1.54	0.674
0–999	1,095 (42.2)	131 (44)		
1,000–4,999	1,298 (50)	149 (50)		
10,000+	25 (1)	3 (1)		
5,000–9,999	178 (6.9)	15 (5)		
Main caregiver			Chisq. (4 df) = 5.24	0.263
Spouse	1,019 (39.3)	113 (37.9)		
Children and other immediate family	19 (0.7)	6 (2)		
Relatives	33 (1.3)	4 (1.3)		
Others	1,434 (55.2)	164 (55)		
None	91 (3.5)	11 (3.7)		

The table above summarizes the distribution of different factors among those with no unintentional injuries (No UI) and those with unintentional injuries (UI). The test statistics and *p*-values indicate the level of significance, with a *p*-value less than 0.05 considered statistically significant.

Table 2 showed the factors associated with unintentional injuries (UI) in an older adult population, focusing on age, gender, marital status, mental health, alcohol consumption, exercise frequency, hypertension, diabetes, main caregiver, and area of residence, using crude and adjusted odds ratios (OR) with 95% confidence intervals (CI) to assess these associations. Significant findings include a higher adjusted odds of UI among individuals with anxiety and depression (adj. OR = 1.92, 95% CI: 1.42–2.6, *p* < 0.001), alcohol consumers (adj. OR = 1.46, 95% CI: 1.05–2.03, *p* = 0.023), and those residing in rural areas (adj. OR = 1.38, 95% CI: 1.05–1.82, *p* = 0.02). Additionally, females showed a significantly higher adjusted odds of UI compared to males (adj. OR = 1.38, 95% CI: 1.03–1.84, *p* = 0.029). Exercise frequency was also significant, with individuals exercising 1–2 times per week having a reduced adjusted odds of UI (adj. OR = 0.46, 95% CI: 0.22–0.98, *p* = 0.044). Other factors such as age, marital status, hypertension, diabetes, and main caregiver did not show significant associations with UI.

**Table 2:** Logistic regression for factors associated with unintentional injuries in older adult population.

	Crude OR (95%CI)	Adj. OR (95%CI)	*P*(Wald’s test)	*P*(LR-test)
Age group: ref. = 60–64				0.522
65–69	1.04 (0.73,1.48)	1.05 (0.73,1.5)	0.801	
70–74	1.15 (0.81,1.65)	1.21 (0.83,1.75)	0.317	
75–79	1.31 (0.86,2)	1.33 (0.86,2.07)	0.204	
80+	1.33 (0.91,1.96)	1.37 (0.89,2.1)	0.148	
Gender: Female vs. Male	1.2 (0.94,1.53)	1.38 (1.03,1.84)	0.03	0.029
Marital status: Ref. = Married				0.649
Unmarried	1.42 (0.49,4.12)	0.77 (0.2,2.99)	0.704	
Widowed	1.22 (0.92,1.61)	1.05 (0.74,1.48)	0.781	
Divorced	1.8 (0.79,4.09)	1.72 (0.72,4.13)	0.224	
Anxiety and depression: Yes vs. No	2 (1.49,2.68)	1.92 (1.42,2.6)	< 0.001	< 0.001
Alcohol consumption: Yes vs. No	1.14 (0.86,1.52)	1.46 (1.05,2.03)	0.023	0.025
Exercise frequency: Ref.= > 6 times				0.138
3–5 times	0.81 (0.47,1.41)	0.79 (0.45,1.38)	0.413	
1–2 times	0.52 (0.25,1.09)	0.46 (0.22,0.98)	0.044	
<1 time	1.21 (0.46,3.14)	1.02 (0.39,2.7)	0.962	
None	1.18 (0.91,1.53)	1.06 (0.8,1.41)	0.667	
Hypertension: Yes vs. No	1.1 (0.85,1.41)	1.02 (0.79,1.34)	0.856	0.857
Diabetes: Yes vs. No	1.34 (0.94,1.92)	1.38 (0.94,2.02)	0.096	0.105
Main caregiver: Ref. = Children				0.396
Spouse	0.97 (0.75,1.25)	1.16 (0.86,1.56)	0.329	
Relatives	2.76 (1.09,7.01)	3.07 (0.94,9.99)	0.063	
Others	1.06 (0.37,3.03)	1.05 (0.36,3.07)	0.933	
None	1.06 (0.55,2.02)	1.26 (0.64,2.45)	0.504	
Area: Rural vs. Urban	1.25 (0.97,1.6)	1.38 (1.05,1.82)	0.02	0.019

The table summarizes the associations of various factors with unintentional injuries, showing crude and adjusted odds ratios (OR) with 95% confidence intervals (CI), Wald’s test *p*-values, and likelihood ratio (LR) test *p*-values. A *p*-value less than 0.05 is considered statistically significant.

## 4 Discussion

The findings of this study reveal several important associations between various factors and the likelihood of unintentional injuries (UI) in an older adult population. Mental health, particularly the presence of anxiety and depression, emerged as a significant factor associated with of UI. This is consistent with existing literature which suggests that mental health conditions can impair cognitive and physical functioning, increasing the risk of accidents and injuries. The adjusted odds ratio (OR) for individuals with anxiety and depression was 1.92, indicating nearly double the risk of UI compared to those without these conditions. This highlights the need for comprehensive mental health support and interventions to mitigate this risk. These findings are consistent with previous research indicating that mental health disorders are not only significant contributors to morbidity but also have a profound impact on the physical safety of the older adult (14–16).

Gender differences were also notable, with females having a significantly lower adjusted odds of UI compared to males (adj. OR = 1.38). This could be due to various socio-cultural and biological factors that influence the activity levels and risk behaviors differently between genders, which aligns with some studies suggesting that older adult women are at a higher risk due to gender-specific health factors (17, 18). Further research is needed to explore these gender-specific dynamics and develop targeted prevention strategies.

Alcohol consumption was another significant factor, with alcohol consumers showing higher adjusted odds of UI (adj. OR = 1.46). Alcohol can impair judgment, coordination, and reaction times, all of which are critical for preventing injuries (19, 20). This finding underscores the importance of monitoring and managing alcohol intake among the older adult to reduce the incidence of UI.

The study also found that residing in rural areas was associated with higher adjusted odds of UI (adj. OR = 1.38). Rural areas may present unique challenges such as limited access to healthcare, fewer community resources, and hazardous living conditions that contribute to a higher risk of injuries, echoing the similar concerns about the rural healthcare system’s ability to manage mental health issues effectively (5, 21). This finding suggests a need for tailored interventions in rural settings to address these specific risks.

Exercise frequency was inversely related to the risk of UI, with those exercising 1–2 times per week having reduced odds of injuries (adj. OR = 0.46). Regular physical activity is known to improve balance, strength, and overall physical fitness, which can help prevent falls and other injuries (22). However, the protective effect was significant only at moderate levels of exercise, indicating that even a small amount of regular physical activity can be beneficial (23).

Other factors, including age, marital status, hypertension, diabetes, and main caregiver, did not show significant associations with UI. This could be due to a variety of reasons, including the possibility that these factors may influence UI risk through more complex mechanisms not captured in this study, or that their effects are mediated by other variables.

Beyond confirming established associations between mental health and injury risks, our study highlights the compounded vulnerabilities in rural Guangxi. The interplay between limited healthcare access and mental health underscores the necessity for integrated care models that simultaneously address psychological well-being and injury prevention. Additionally, our findings suggest that even moderate levels of physical activity can significantly reduce injury risks, advocating for community-based exercise programs tailored to the older adult.

The study on the link between mental health disorders and unintentional injury risks among the older adult in Guangxi, China, underscores significant public health implications. Key findings advocate for integrating mental health care into injury prevention strategies for the aging population. Policy implications stress the necessity for integrated care models that include mental health screening and treatment within primary healthcare (24), enhancing accessibility and quality of mental health services in rural areas to mitigate associated injury risks (25), and promoting public health campaigns to raise awareness about the importance of mental health maintenance (26). Practice recommendations call for routine mental health assessments during medical check-ups for the older adult (27), training healthcare professionals to recognize and manage the correlation between mental health and injury risk (28), and developing community support structures that provide social support and reduce injury risk (29). Moreover, there is a need for longitudinal research to understand the causal relationships (30) and for studies focusing on tailored interventions that address both mental health and injury prevention, particularly in rural settings (31).

## 5 Limitations

While this study offers valuable insights into the relationship between mental health and unintentional injury risks among the older adult in Guangxi, China, it is important to acknowledge several limitations. The cross-sectional design restricts the ability to draw causal inferences, with longitudinal data needed to establish a clearer temporal and causal relationship between mental health issues and injury risks. The reliance on self-reported data may introduce recall bias and affect data accuracy, whereas objective measures and clinical evaluations would provide more reliable information. Cultural nuances and language differences might also influence the interpretation of survey questions, potentially leading to underreporting or misclassification of mental health symptoms. Despite efforts to control for various confounders, unmeasured variables such as the severity of depression or anxiety and the presence of other comorbidities might influence the outcomes. While this study provides valuable insights into the older adult population in Guangxi, caution should be exercised when generalizing these findings to other regions due to Guangxi’s unique socio-economic and cultural characteristics. Future research should aim to include diverse regions to enhance generalizability. Additionally, the National Health Service Survey did not categorize specific types of unintentional injuries, which prevented a more detailed analysis of injury types and their associated factors.

## 6 Conclusion

In conclusion, the study underscores the multifaceted nature of unintentional injuries in the older adult, highlighting key areas such as mental health, alcohol consumption, gender differences, rural residency, and exercise frequency. These findings can inform the development of targeted interventions and policies aimed at reducing the incidence of UI in this vulnerable population. Future research should continue to explore these associations, considering potential mediating factors and the efficacy of specific preventive measures.

## Data Availability

The raw data supporting the conclusions of this article will be made available by the authors, without undue reservation.
